# Damage Identification of Fiber-Reinforced Composite Thin Plate by Curvature Modal Shape Scanning Method

**DOI:** 10.3390/ma18112431

**Published:** 2025-05-22

**Authors:** Yougle Chang, Qi Zhao, Hao Han, Xiaodi Zhao, Lingyao Qin, Xiaoye Li, Liyan Wu, Hui Li

**Affiliations:** 1 Shijiazhuang Division of PLAA Infantry College, Shijiazhuang 050011, China; 2 School of Mechanical Engineering & Automation, Northeastern University, Shenyang 110819, China; lihui@mail.neu.edu.cn

**Keywords:** fiber-reinforced composite thin plate, two-dimensional five-spot-tripling surface smoothing method, damage location detection, curvature modal shape scanning method, damage localization index, experimental study

## Abstract

The damage location detection of the fiber-reinforced composite thin plate (FCTP) is studied through the curvature modal shape scanning method (CMSSM), utilizing the advantages of the sensitivity of curvature modal shapes to local stiffness changes and the high measurement accuracy of a laser vibrometer. Firstly, our research begins with the construction of a laser scanning frame model for the FCTP. Subsequently, during the analysis of modal shape data extraction principles, the two-dimensional five-spot-tripling surface smoothing method is developed, so that the quantitative index for damage location detection of the FCTP, i.e., the damage localization index, can be derived. The operating deflection shapes of the FCTP at different natural frequencies are obtained, and the self-developed laser scanning vibration testing system is employed to scan and measure the vibration. Then, a TC500 fiber/epoxy composite plate is utilized as an experimental object to perform a damage identification experiment. It has been proven that this approach can detect the fiber breakage location of the FCTP with high accuracy. Finally, the influence of parameters such as boundary constraint, excitation level, and laser scanning rate on the damage detection results is also discussed. Through studies on influencing parameters, practical guidance is provided for the application of the damage identification approach of the FCTP.

## 1. Introduction

Fiber-reinforced composite structures have excellent properties, including high specific strength and modulus, good thermal stability, high bearing capacity, and low weight. They are extensively applied in naval vessels, the weapons industry, aeronautics, and astronautics [1,2]. Often subject to high temperatures, high pressure, excessive vibration and noise, supersonic gas, and other harsh conditions, such composite materials and structures are likely to produce defects, such as fiber fracture, matrix cracking, and delamination [3,4]. For example, aircraft composites [5] are exposed to impact damage caused by foreign objects such as dropped tools, birds, or fragments of stones from the runway. It has been reported that impacts cause about 80% of all damage [6]. This in-service damage progressively undermines the mechanical integrity of the structures and sometimes even leads to severe accidents [7,8]. Impact damage is not easy to detect because the damage is often invisible to visual inspection, e.g., interply and interplay damage. Concurrently, aircraft structures endure cyclic stress from operational fatigue loads combined with extreme environmental stressors such as ultraviolet degradation, thermal cycling, and humidity infiltration. As a result, the condition of composite materials affected by impact-induced damage can deteriorate. This environmental exposure can further reduce the longevity and endurance of fiber-reinforced composite structures [9]. Therefore, only by detecting fiber breakage positions of such composite structures in time, repairing them as soon as possible, and restoring the carrying capacity and safety of the structure can the full service life of the structure be extended and the occurrence of catastrophic accidents reduced.

For a long time, much research has been carried out on the damage identification of metal and composite structural parts such as beams and plates, and some research results have been achieved. Compared with the current nondestructive testing technology commonly used at home and abroad, the vibration-based structural damage detection method is a globally adopted structural damage detection and evaluation technology [10,11] that can detect and determine the overall performance of the structure and locate the damage. Some typical vibration-based nondestructive testing methods, such as the frequency response method [12], strain energy method [13], lumped mass approach [14], and vibration power flow [15], have also been applied to the damage detection in composite structures. At the same time, the use of modal shapes for damage detection is widespread. For instance, Roy et al. [16] employed a continuous scanning laser vibrometer to monitor damage in fiber-reinforced composite plates with delamination. They proposed a damage identification and localization method based on modal shapes, which requires obtaining the modal shapes of both healthy and damaged composite plates separately and using their differences for damage localization. Vanlanduit et al. [17] considered the effects of background noise and nonlinear distortions on damage detection and introduced a method combining linear and nonlinear approaches, utilizing a continuous scanning laser vibrometer to acquire modal shape data. Additionally, the Coordinate Modal Assurance Criterion (COMAC) proposed in reference [18] was adopted to locate delamination damage in glass fiber/polyester composite thin plates.

However, in 1991, Pandey and Biswas et al. [19] successfully detected damage in the cantilever beam and the simply supported beam based on the curvature of mode shapes before and after structural damage, achieving excellent results. Existing research has found that curvature modal shapes are more sensitive than frequencies and mode shapes [20] and can accurately locate damage. However, this method requires signals from healthy structures as a benchmark. For example, Govinddasamy et al. [21] used the curvature modal shapes technique for the detection of large fiber breakage damage features in fiber/polyester composite thin plates. This method required combining experiments with FEA, which limited its practical application. Pacheco et al. [22] detected damage features on the surface of a relatively complex composite thin-wall beam by using the curvature modal shapes method, which required information from the healthy structure and was baseline-free.

Ratcliffe [23] developed the gapped smoothing method for one-dimensional beam structures, which operated solely on data obtained from the damaged beam structure to detect stiffness variability in the structural beam. Initially, only the resonant data such as modal shape data were used to detect the local stiffness variability in the gap steel beam. Ratcliffe successfully used this method to detect delamination in composite beams [24]. Later, broadband data were used by employing the curvature modal shapes obtained from the FRF data. This method remarkably improved the sensitivity of structural variability detection [25]. This method enables damage localization by analyzing signals from the damaged structure alone, eliminating the need for healthy-state parameter comparisons. Yang et al. [26] combined this method with cross-correlation functions and wavelet transforms, proposing a combined localization method for damage of composite structures. Yoon et al. [27] also attempted to develop this interval smoothing method into a two-dimensional approach. However, the smoothing method used had poor accuracy, and they did not adopt the operational deflection shapes method for detection.

Although extensive research has been conducted by scholars and researchers on the damage identification of fiber-reinforced composites, most of the current vibration-based nondestructive testing methods need to measure the signal of the structure in health, and it is difficult to effectively detect the structure without the health signal. Some scholars have proposed using a two-dimensional interval smoothing method to obtain the healthy signal, and the smoothing method has poor accuracy. This significantly limits its widespread application in engineering structures.

In this research, utilizing the advantages of the sensitivity of curvature modal shapes to local stiffness changes and the high measurement accuracy of laser vibrometers, the curvature modal shape scanning method (CMSSM) is employed to study the damage location detection of the fiber-reinforced composite thin plate (FCTP). Firstly, our research begins with the construction of a laser scanning frame model for the FCTP. Subsequently, during the analysis of modal shape data extraction principles, the two-dimensional five-spot-tripling surface smoothing method is developed, so that the quantitative index for damage location detection of the FCTP, i.e., the damage localization index, can be derived. Then, an electromagnetic exciter is employed to excite the FCTP, and its operating deflection shapes at different natural frequencies are obtained, and the self-developed laser scanning vibration testing system is employed to scan and measure the vibration. Then, a TC500 fiber/epoxy composite plate is utilized as an experimental object, and a damage identification experiment is performed. Finally, the influence of parameters such as boundary constraint, excitation level, and laser scanning rate on the damage detection results is also discussed. The parameters of constrained boundary conditions, excitation level, and laser scanning rate have significant influences on damage location detection, and identifying average damage localization indexes (ADLIs) can be quantitatively analyzed. Through studies on influencing parameters, practical guidance is provided for the application of the damage identification approach of the FCTP. It has been proven that this above approach can detect the fiber breakage location of the FCTP with high accuracy, and this method and the related experimental system can provide a new idea for damage detection and identification of composite structures.

## 2. Damage Location Detection Principle of the FCTP Based on the CMSSM

In this part, a method based on the CMSSM is proposed and the theoretical principle of damage location detection of the FCTP is introduced in detail.

### 2.1. To Obtain Modal Shape of the FCTP Based on Laser Scanning Method

**Figure 1:**
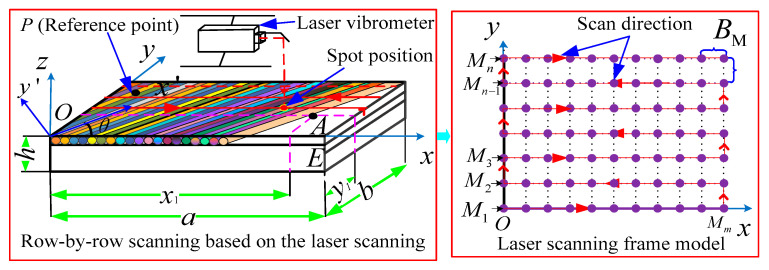
The laser scanning frame model of the FCTP.

First, Figure 1 shows a laser scanning frame model established of the FCTP, which was mainly obtained through row by row scanning of the FCTP by a single-point laser vibrometer. Assuming *O*-*xyz* as the global coordinate system, the x axis is located on the upper surface of the structure, and the angle between the fiber direction $x^{\prime }$ and the x axis direction of the whole coordinate system is θ. The length of the FCTP is *a*, b for the width, *h* for the thickness, *ρ* for the density, *m*_s_ for the quality.

Assuming that the laser point of the laser vibrometer can be scanned in a straight line, and the scanning path of the laser point divides the FCTP into $M_{1},M_{2},\cdots M_{m}$ lines along the *x* direction, m,n is the maximum number of rows and columns corresponding to the line scan path, respectively, the numbers of measure points in the *x* direction and the *y* direction are $K_{n},\;K_{m}$, *i* and *j* are sequence numbers of any scanning point *A*(*i*, *j*) in the directions of *x* and *y*. At the same time, is it assumed that the intervals of scanning between each row and each column are equal, which can be represented by the interval distance $B_{M}$. In addition, setting up an acceleration sensor at a fixed point *P* on the reference position of the FCTP, the test data will be extracted together with the laser scanning data.

When scanning row by row, the number of points Kn required to be loaded in the frame model along each row in the *x* direction can be expressed as:

(1) $$K_{n}=\frac{a+B_{M}}{B_{M}}$$

Then, for each column in the *y* direction, the number of points $K_{m}$ required to be loaded in the frame model is

(2) $$K_{m}=\frac{b+B_{M}}{B_{M}}$$

Assuming scanning the FCTP with a laser at the scanning speed of *v*_s_, when the laser point scans the measurement point *A*, the time wave of the response signal obtained by the laser vibrometer is ${\dot{\tilde{z}}}_{i,j}(t)$, and the FFT transformation of ${\dot{\tilde{z}}}_{i,j}(t)$ can obtain the frequency response of ${\dot{\tilde{z}}}_{i,j}(\omega )$. At the same time, the response signal $z_{P}(t)$ at the reference point *P* is transformed by FFT, and it is assumed that the frequency response is $z_{P}(\omega )$, then the ratio of the two is defined as the transfer rate function $\gamma_{i,j}(\omega )$, which can be expressed as

(3) $$\gamma_{i,j}(\omega )=\frac{{\dot{\tilde{z}}}_{i,j}(\omega )}{z_{p}(\omega )},i=1,2,\cdots K_{n},j=1,2,\cdots K_{m}$$

where $\gamma_{i,j}(\omega )$ represents the transfer rate function of the location of an arbitrary point measured by the laser vibrometer under the line scanning mode.

When an FCTP is excited at a certain resonant frequency ωr, according to the resonance theory, when the modes of each order have no coupling, Formula (3) can be approximated to

(4) $$\gamma_{i,j}(\omega )\approx \frac{\phi_{(i,j)r}}{\phi_{pr}}={\bar{\phi }}_{(i,j)r},i=1,2,\cdots K_{n},j=1,2,\cdots K_{m}$$

where $\phi_{(i,j)r}$ and $\phi_{Pr}$ are the component values of the order *r* modal shape at the arbitrary scanning point *A*(*i*, *j*) and the fixed reference point *P*.

But in engineering practice, for reducing the influence of external noise, the transfer rate function $\gamma_{i,j}(\omega )$ is generally obtained by dividing cross-power spectrum $G_{(i,j)P}(\omega )$ which is gained by scanning the response of the measuring point *A* and the fixed reference point by the auto-power spectrum $G_{PP}(\omega )$ of the response of the fixed reference point, therefore Formula (4) can be transformed as

(5) $$\gamma_{i,j}(\omega )=\frac{{\dot{\tilde{z}}}_{i,j}(\omega ){z^{*}}_{P}}{z_{P}(\omega ){z^{*}}_{P}}=\frac{G_{(i,j)P}(\omega )}{G_{PP}(\omega )}i=1,2,\cdots K_{n},j=1,2,\cdots K_{m}$$

where $G_{(i,j)P}(\omega )$ is cross-power spectrum of any scanning point *A* responses and fixed reference point *P* response, while $G_{PP}(\omega )$ is the auto-power spectrum of the fixed reference point *P* response. In addition, $z^{*}{\left(\omega \right)}_{P}$ is the conjugate value of $z{\left(\omega \right)}_{P}$.

Because the vibration response of the same reference point is used as the reference signal, the transfer rate function ensures the phase relation of different response measuring points relative to the reference points, and the influence of the change of different measuring groups’ operation conditions can basically be eliminated. In addition, under resonance conditions where the excitation frequency matches the structure’s natural frequency $\omega_{r}$ of a certain order, we can obtain the amplitude and phase of the transfer rate function $\gamma_{i,j}$ at the natural frequency $\omega_{r}$ through Formula (5), and then obtain the vibration size and direction information corresponding to a scanning measuring point through its amplitude and phase. It needs to be explained that, due to the limited space, Ref. [28] can be used to clear the method of extracting the corresponding time domain data of different laser scanning points and reference points. Once the above data are obtained, we can obtain the transfer rate function concerned by spectrum analysis technology.

### 2.2. Extraction of Curvature Modal Shape of the FCTP

First, with the regularization processing of the transfer rate function $\gamma_{i,j}$, we can obtain:

(6) $$\varphi_{i,j}(\omega )=\gamma_{i,j}(\omega )\sqrt{K_{m}\cdot K_{n}/\sum_{i}^{K_{n}}\sum_{j}^{K_{m}}\left|{\gamma^{2}}_{i,j}(\omega )\right|}$$

where $\varphi_{i,j}(\omega )$ is the transfer rate function after the regularization processing at any measuring point *A*(*i*, *j*).

Supposing the FCTP is formed by uniformly varying fiber materials, the greater the stiffness changes at a certain position, that is, the greater the degree of damage, the more obvious the singularity of curvature will be in this area [23]. Therefore, we can realize the damage location according to the above principle, and we generally use the two-dimensional center difference method to approximate the curvature modal shape at the measuring point *A*(*i*, *j*):

(7) $$Z_{i,j}(\omega )=\left(\varphi_{i+1,j}(\omega )+\varphi_{i-1,j}(\omega )-2\varphi_{i,j}(\omega )\right)/B_{M}^{2}+\left(\varphi_{i,j+1}(\omega )+\varphi_{i,j-1}(\omega )-2\varphi_{i,j}(\omega )\right)/B_{M}^{2}$$

### 2.3. Determination of Damage Location Based on the Two-Dimensional Five-Spot-Tripling Surface Smoothing Method

On the basis of the five-spot-tripling smoothing theory, this section generalizes it to two dimensions, and it is called the ‘two-dimensional five-spot-tripling surface smoothing method’, which has many outstanding advantages, such as fast computation and accurate location of damage, and the following is a detailed description of its positioning principle.

**Figure 2:**
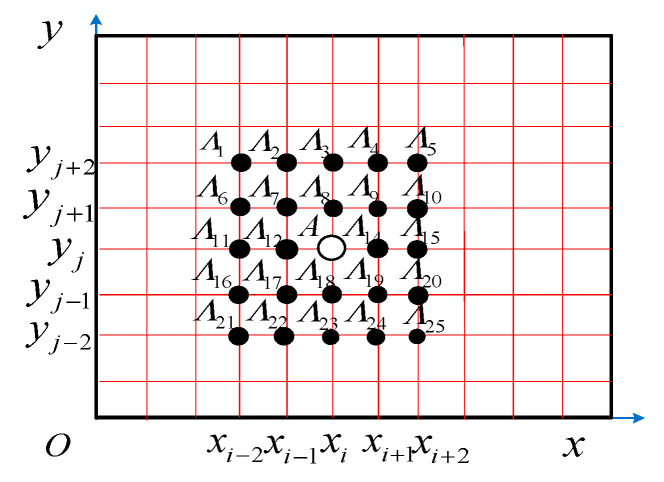
Scanning points and their equidistant points when applying the two-dimensional five-spot-tripling surface smoothing method.

First of all, set up the coordinate value as $x_{i},y_{j}$ of any scanning point *A*(*i*, *j*) in the *x* and *y* directions, as shown in Figure 2, and suppose that any isometric measuring point is $\Lambda_{1},\Lambda_{2},\cdots \Lambda_{12},A,\Lambda_{13},\cdots ,\Lambda_{25}$ in *x* and *y* directions (where the total number of *A* points is 25, and the measuring point Λ is used for the parameter estimation of the measuring point A), and the coordinate values of each measuring point are shown in Figure 2. According to the five-spot-tripling smoothing theory, in order to obtain a smoothed curvature modal shape, the curvature modal shapes obtained by Equation (7) are expressed as a mathematical form:

(8) $$Z_{\alpha ,\beta }(\omega )=\sum_{\tilde{i}=0}^{3}\sum_{\tilde{j}=0}^{3}C_{\tilde{i},\tilde{j}}\alpha^{\tilde{i}}\beta^{\tilde{j}}$$

where α,β, and $C_{i,j}$ are respectively the relative position coefficient and the fitting coefficient when we perform the three five-point operations of any isometric measuring point Λ relative to the measuring point *A*(*i*, *j*). Since the coefficient $C_{i,j}$ is known at any grid point and the curvature is calculated by Equation (7). α and β can be expressed as:

(9) $$\alpha =\frac{x_{\Lambda }-x_{A}}{B_{M}}$$

(10) $$\beta =\frac{y_{\Lambda }-y_{A}}{B_{M}}$$

Through Formulas (9) and (10), we can calculate α,β=−2,−1,0,1,2, then the least square method is used to determine the fitting coefficient $C_{i,j}$ of the two-dimensional five-spot-tripling smoothing theory, supposing that the least square function *δ* is as follows:

(11) $$\delta =\sum_{\alpha =-2}^{2}\sum_{\beta =-2}^{2}{\left[\sum_{\tilde{i}=0}^{3}\sum_{\tilde{j}=0}^{3}C_{\tilde{i},\tilde{j}}\alpha^{\tilde{i}}\beta^{\tilde{j}}-Z(\alpha ,\beta )\right]}^{2}$$

Thus, solving $C_{\tilde{i},\tilde{j}}(\tilde{i},\tilde{j}=0,1,2,3)$ and substituting it into Formula (8) gives the two-dimensional five-spot-tripling smoothing formula:

(12) $${\bar{Z}}_{\alpha ,\beta }(\omega )=\sum_{\tilde{i}=0}^{3}\sum_{\tilde{j}=0}^{3}C_{\tilde{i},\tilde{j}}{\left(\alpha \right)}^{\tilde{i}}{\left(\beta \right)}^{\tilde{j}}$$

where $\bar{Z}(\alpha ,\beta )$ is Z(α,β), the smooth curvature modal shape obtained after two-dimensional five-spot-tripling smoothing operation.

When the measuring point *A*(*i*, *j*) is on the corners or edges of the FCTP, the construction of the coefficient $C_{\tilde{i},\tilde{j}}$ is slightly changed to match the neighboring points (see Figure 2). In order to enable least squares estimation or explicit parameters, the number of parameters should be less than or the same as the number of neighboring points.

Finally, as described in reference [29], the damage localization index is derived by calculating the absolute difference between the curvature modal shape values and their smooth curvature modal shape values (obtained using a two-dimensional five-spot-tripling surface smoothing method) at identical points. And the damage localization index formula is given as follows:

(13) $$\delta_{i,j}(\omega )=\left|Z_{i,j}(\omega )-{\bar{Z}}_{i,j}(\omega )\right|$$

To address potential inaccuracies in damage localization when the damage is located near a modal node (a point with minimal vibration amplitude in a specific mode shape) of the FCTP, the damage localization index $\delta_{i,j}(\omega )$ obtained under excitations at different natural frequencies can be averaged to enhance localization accuracy. The averaged damage localization index ${\bar{\delta }}_{i,j}(\omega )$ is then expressed as:

(14) $${\bar{\delta }}_{i,j}(\omega )=\frac{1}{\Pi }\sum_{\chi =\mu }^{\nu }\left|Z_{i,j}(\omega_{\chi })-{\bar{Z}}_{i,j}(\omega_{\chi })\right|$$

where μ and ν denote the mode orders corresponding to the lowest-order natural frequency and highest-order natural frequency, respectively. The parameter Π presents the difference in mode orders between the natural frequencies while used for averaging, defined as $\Pi =\left|\mu -\nu \right|+1$.

## 3. Damage Identification Procedure of FCTP

In Section 1, this paper clarifies the principle of damage identification of FCTP based on the laser scanning method. This part proposes the specific procedure of damage location detection, which can be partitioned into the following six core steps:

1. Accurately determining the modal natural frequencies of the damaged FCTP.

In order to accurately determine the modal natural frequency results of the damaged FCTP, firstly the natural frequencies can be roughly obtained through hammer excitation modal testing using the single-point excitation and single-point response method, or we can theoretically calculate by finite element analysis to obtain natural frequencies and modal shapes within the targeted frequency range as reference data. Then, according to these frequency results, we can determine the sweep frequency range of each mode of the damaged composite plate. Finally, by identifying the response peak in the three-dimensional waterfall of each mode, we can accurately obtain modal natural frequencies of the damaged FCTP.

2. Exciting the FCTP to the resonance state of the sufficiently energized, conducting laser scanning tests and acquiring the response signals.

The vibration exciter’s driving frequency must be precisely calibrated to match a specific-order natural frequency of the damaged FCTP, with sustained constant-frequency and fixed-amplitude excitation. To effectively capture modal operational deflection shapes, the prescribed excitation force amplitude must exceed the threshold required to induce sufficient resonant modal response corresponding to the targeted vibrational mode order.

3. Evaluating the quality of the resonance response data based on the coherence function.

Since the quality of resonance response signal testing significantly impacts the accuracy of damage location detection, it is essential to evaluate the quality of resonance response data using the coherence function. The coherence function between each response point on the FCTP and the reference point can be expressed as

(15) $${\lambda^{2}}_{(i,j)P}(\omega )=\frac{{\left|G_{(i,j)P}(\omega )\right|}^{2}}{G_{i,j}(\omega )G_{P}(\omega )},i=1,2,\cdots K_{n},j=1,2,\cdots K_{m}$$

In this formula, λ≥0.85, and it is considered that the response signal is completely caused by the excitation signal issued by the electromagnetic exciter. This condition indicates negligible environmental noise interference and high signal quality, thereby validating the data for subsequent modal shape identification.

4. Applying windowing and filtering processes to the resonance response signals.

**Figure 3:**
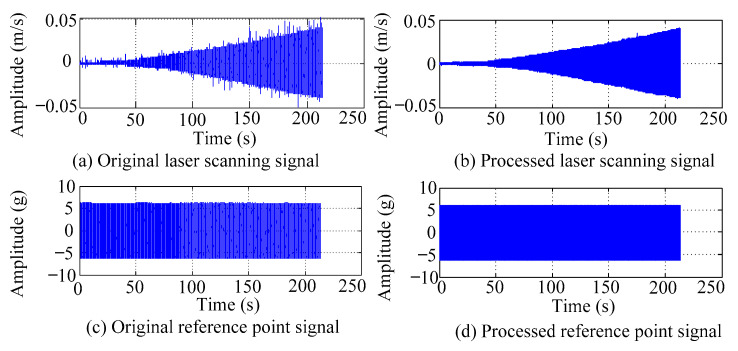
The time-domain waveforms of reference points and laser scanning points under the first resonance state.

To reduce spectral energy leakage, windowing should be applied to the resonance response signal, and the Hanning window is recommended due to its high amplitude recognition accuracy. Additionally, a low-pass filter can be employed to eliminate background noise components in the time domain signal. Taking the acquisition of the first resonance response signal of the FCTP as an example, Figure 3 illustrates the time domain waveforms of the reference point and the first-row laser scanning point before and after windowing and filtering processing.

5. Normalization process and obtain the curvature modal shape of some order.

After windowing and filtering the resonance response data of some order, they are substituted into Equation (5) to obtain the transfer rate function $\gamma_{i,j}$. Then, the transfer rate function $\gamma_{i,j}$ is normalized through Equation (6), which is transformed into a dimensionless expression and becomes pure quantity (the normalized modal amplitude retains only relative significance, as its absolute value is meaningless). Then, the normalized modal amplitude is brought into Equation (7) and the curvature modal shape of the damage composite plate is calculated.

6. Determine the damage location of the FCTP.

Repeat step 1 to step 5 to obtain the curvature modal shape corresponding to different orders of the damage composite plate of interest. Then, the two-dimensional five-spot-tripling surface smoothing method is used to obtain the corresponding smooth curvature pattern value of each point, and then the above parameters are brought into Equation (15) to obtain the average damage index. Finally, based on the dimensions of the FCTP, the laser scanning frame model is generated for the loading date. The computed ADLI is mapped to the coordinates of each scanning measurement point, thereby enabling the identification of the damage location.

7. The data processing and analysis steps.

**Figure 4:**
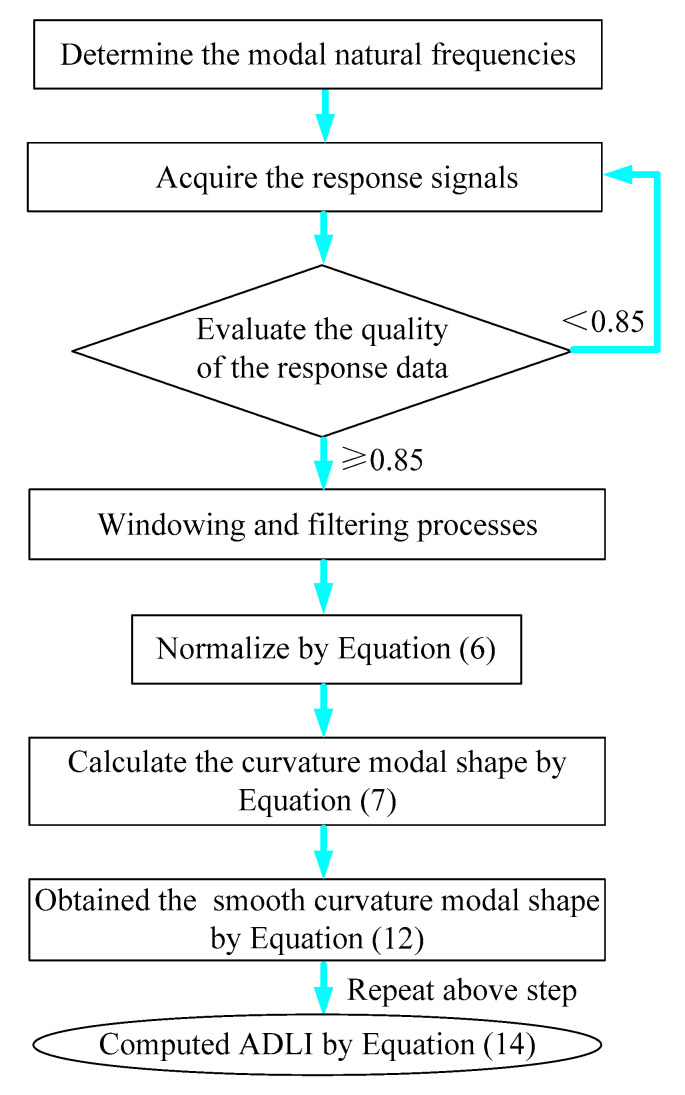
The algorithm flowcharts of TMATLAB 2012b.

According to the above theoretical principles, based on steps 1–6, after repeated experiments and continuous summary, the following MATLAB 2012b data processing and analysis steps can be obtained. The key MATLAB 2012b program is shown below (Figure 4):

Calculate the curvature modal shape:

*for i = 2: K_n_* − *1*

*for j = 2: K_m_* − *1*

*zzfa = zzz(i, j);*

*zzfa_up = zzz(i + 1, j);*

*zzfa_right = zzz(i, j + 1);*

*zzfa_down = zzz(i* − *1, j);*

*zzfa_left = zzz(i, j* − *1);*

*zzqlv(i* − *1, j* − *1) = (zzfa_up + zzfa_down* − *2 × zzfa)/(B_M_* × *B*_M_*) + (zzfa_right + zzfa_left − 2 × zzfa)*

*/(B_M_ × B_M_);*

*end*

*end*

Obtain the smooth curvature modal shape:

*for i = 2: K_n_* − *1* − *2*

*for j = 2: K_m_* − *1* −*2*

*A = [1, (j* − *1) × B_M_, (i* − *2) × B_M_;*

*1, (j* − *0) × B_M_, (i* − *2) × B_M_;*

*1, (j* − *0) × B_M_, (i* − *1) × B_M_;*

*1, (j* − *0) × B_M_, (i* − *0) × B_M_;*

*1, (j*− *1) × B_M_, (i* − *0) × B_M_;*

*1, (j*− *2) × B_M_, (i* − *0) × B_M_;*

*1, (j*− *2) × B_M_, (i* − *1) × B_M_;*

*1, (j*− *2) × B_M_, (i* − *2) × B_M_; ];*

*B = [zzqlv(i* − *1, j); zzqlv(i* − *1, j + 1); zzqlv(i, j + 1);zzqlv(i + 1,j + 1); zzqlv(i + 1, j); zzqlv(i + 1, j* − *1);*

*zzqlv(i, j* − *1); zzqlv(i* − *1,j* − *1)];*

*X = A\B*

*Gij = [1, (j* − *1) × B_M_,(i* − *1) × B_M_];*

*zzqsmooth(i, j) = Gij × X;*

*end*

*end*

Where *zzz* is the modal shape data, and the other parameters are defined values.

## 4. Case Study

### 4.1. Experimental Object

**Figure 5:**
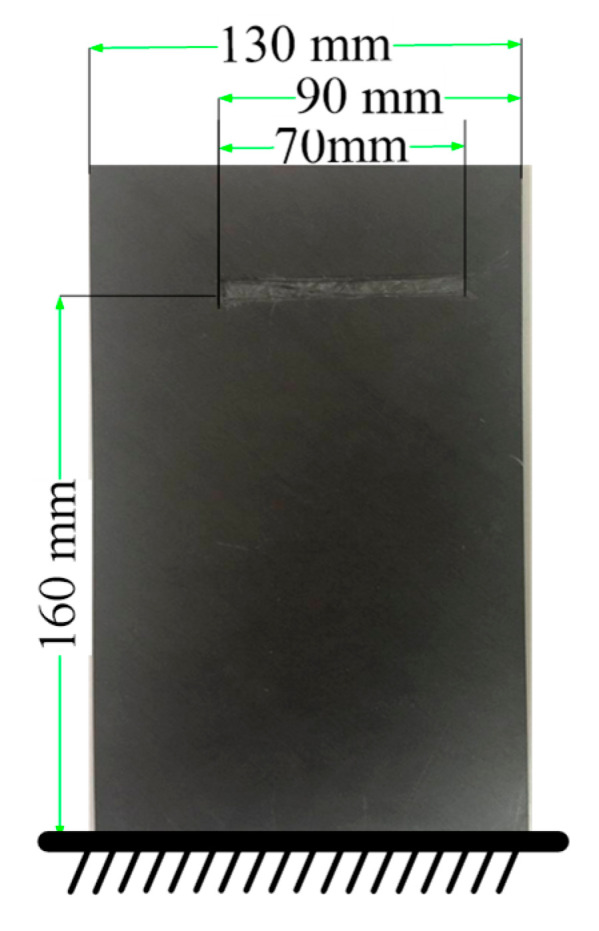
The picture of the FCTP with fiber breakage damage.

A TC500 carbon/resin composite plate with fiber breakage damage is taken as a research object. Figure 5 shows an artificially created crack (produced by damaging the FCTP with a sharp knife, with a damage depth of approximately 20% of the plate thickness). The ‘broken line’ has a length of 70 mm and is located 160 mm away from the cantilever constraint end of the FCTP, with specific positional parameters illustrated in Figure 5.

The composite plate consisted of TC500 carbon fibers (Toray Industries, Tokyo, Japan, 12 K tow, tensile modulus 240 GPa), impregnated with EPOLAM 2024 epoxy resin (Jiangsu Sanmu Gtoup, Jiangsu, China, cured at 120 °C for 2 h, glass transition temperature *T_g_* = 150 °C). The fiber volume fraction was 60 ± 2%. This type of FCTP is symmetrically orthogonally laid, namely [−45/45]_s_, with a total of 20 layers. And each layer has the same thickness and fiber volume fraction. The plate is fixed using the fixture shown in Figure 5, which clamps one edge with a gripping length of 30 mm to simulate cantilever boundary conditions. The constrained FCTP has final dimensions of 200 mm × 130 mm × 2.36 mm (length × width × thickness).

### 4.2. Experimental Equipment

**Figure 6:**
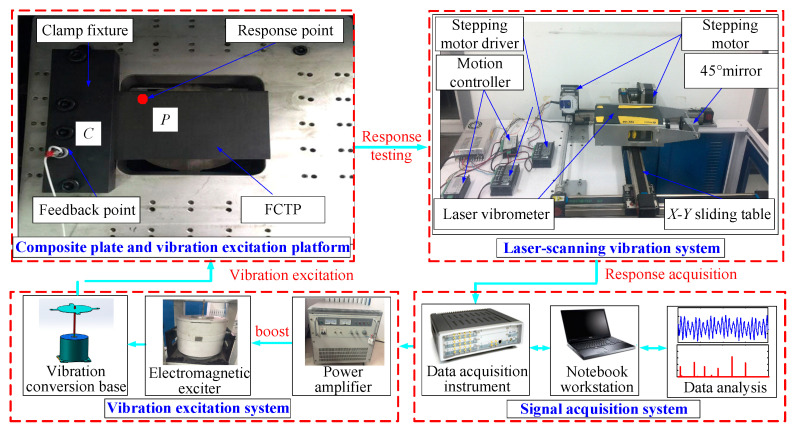
Connection schematic of laser scanning vibration testing system of the FCTP.

**Figure 7:**
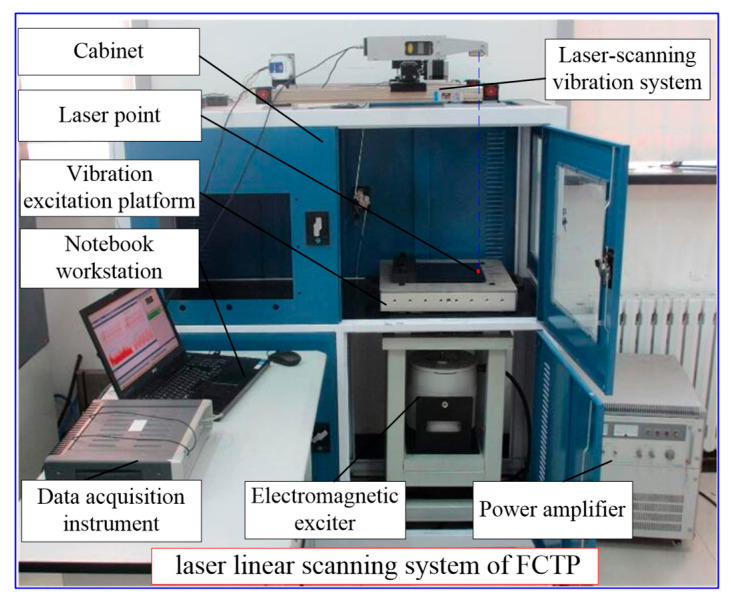
The real picture of damage location measurement of the FCTP.

Figure 6 shows the connection schematic of the laser scanning vibration testing system, and Figure 7 shows the real picture of damage location measurement of the FCTP. The main experimental equipment and instruments include: the laser scanning system, vibration excitation devices, the vibration excitation platform, and the signal acquisition system. Specific models are as follows:

- LMS SCADAS (Siemens, Munich, Germany) 16-channel data acquisition instrument;
- LianNeng JZK-100 (Sinocera Piezotronics Inc., Zhejiang, China) electromagnetic exciter (maximum sinusoidal excitation force: 1000 N) and LianNeng YE5878 power amplifier (rated output power: 1200 W, output voltage: 40 Vrms);
- Polytec PDV-100 (Polytec GmbH, Karlsruhe, Germany) laser vibrometer (frequency range: 0–22 kHz, minimum velocity resolution: 0.05 μm/s; the light source uses a He-Ne laser (λ = 632 nm) with an output power of 1 mW and a beam diameter of 1 mm and does not cause additional damage to TC500);
- A self-developed excitation platform equipped with a B&K4508B accelerometer (Brüel & Kjær, Copenhagen, Denmark) on its surface.

In addition, four holes were designed in the fixture to securely clamp the FCTP. The first three natural frequencies of the FCTP were repeatedly measured under different tightening torques. Through comparing with each other, the best repeatability of frequency values can be reached when the tightening torque is 50 Nm, so this torque value is always used in the measurement. After the repeated testing and comparison, a B&K 4517 lightweight accelerometer is glued firmly at reference point *P* by super glue 502. And this reference point *P* is located on the backside of the FCTP, 30 mm from both the constrained end and the lower free edge.

During the experiment, according to the damage identification procedure of the FCTP in Section 3, a sine sweep excitation signal is first generated by the LMS acquisition system and applied to the FCTP via the electromagnetic exciter. The control software developed based on LabVIEW 2025 is then used to adjust the specific position of the laser spot of the laser vibrometer, while the LMS acquisition system performed real-time acquisition of the response signals obtained by the laser vibrometer. Additionally, the following experimental settings and parameters are selected: (I) row spacing of $B_{M}=10\;\mathrm{mm}$, (II) excitation amplitude of 1100 N, (III) scanning speed of 3 mm/s, (IV) sampling frequency of 3200 Hz, (V) frequency resolution of 0.125 Hz, (VI) application of a Hanning window function to both the excitation and response signals, (VII) sine sweep frequency rate of 1 Hz/s.

### 4.3. Test Results

**Figure 8:**
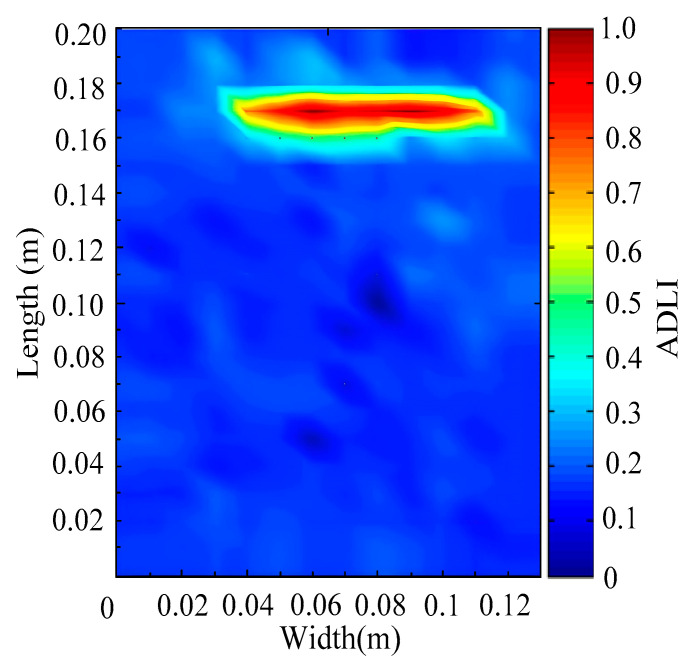
Two-dimensional drawing of the laser scanning frame model when loading the first 5 ADLIs.

**Figure 9:**
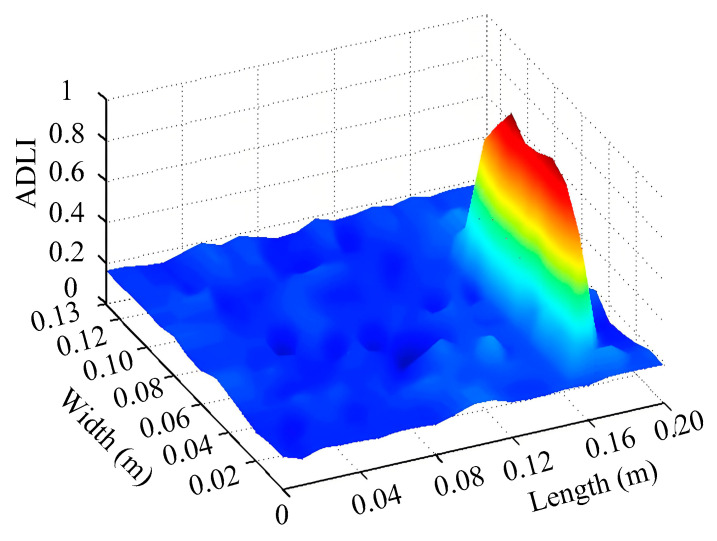
Three-dimensional drawing of the laser scanning frame model when loading the first 5 ADLIs.

Through the sweep frequency testing approach, the natural frequency results can be precisely determined by identifying the peaks in the frequency domain response curve corresponding to a specific mode. Subsequently, the FCTP is excited to resonance at these identified natural frequencies, and the laser scanning vibration testing system is employed to capture the modal shapes associated with each natural frequency. Table 1 presents the first five experimentally measured natural frequencies and modal shapes of the FCTP with damage. Then, the smooth curvature modal shapes for the first five modes are calculated using the two-dimensional five-spot-tripling surface smoothing method implemented in a MATLAB 2012b program, and these results are also included in Table 1. Finally, the damage localization indexes $\delta_{i,j}(\omega )$ of the first five natural frequencies are averaged to obtain the ADLI, which is then mapped onto the laser scanning frame model, with its two-dimensional drawing and three-dimensional drawing illustrated in Figure 8 and Figure 9, respectively.

The analysis of the data in Table 1 reveals that the presence of fiber breakage damage causes discontinuities in the modal shapes of the FCTP, manifested as singular regions near the fiber breakage in the curvature modal shapes. However, while the curvature modal shapes indicate the existence of damage, they are insufficient to precisely locate its specific position.

When the CMSSM proposed in this study is applied for damage identification, both Figure 8 and Figure 9 demonstrate that the ADLI is significantly higher near the fiber breakage compared to other regions. These indicate an alteration in the torsional stiffness at the damaged location and the presence of singularities in the curvature modal shapes, confirming structural damage in this area. The accuracy of the experimental results is proved by comparing the actual damage location with the physical damage location. Thus, the proposed method effectively and accurately identifies the specific damage location in the FCTP.

## 5. Discussion

The accuracy of the damage location index test is influenced by the following factors:

1. Impact assessment of the varied constraint boundary conditions.

Owing to the ubiquitous implementation of the cantilever beam boundary condition of the FCTP in engineering practice, we firstly discuss the influences of varied cantilever boundaries on the test accuracy of modal shape results. In the actual experiment, we employ three different torque values to tighten the four M12 bolts, namely 5 Nm, 30 Nm, and 50 Nm of tightening torque, to simulate three different constraint boundaries. Then, according to the test procedure proposed in Section 3, we can finish the modal shape scanning measure in the row scanning path mode, and the laser scanning rate is set as 3 mm/s with the adjacent row spacing of 10 mm.

Table 2 lists the 3rd curvature modal shapes of the FCTP measured under the three different constraint boundaries. Table 3 lists the 3rd damage localization indexes of the FCTP measured under the three different constraint boundaries. It can be observed that the constraint boundary condition has a great influence on the curvature modal shapes and the damage localization indexes. When the boundary condition is elastic, i.e., the tightening torque value which is related to the constraint boundary is not enough, the curvature modal shapes and the damage localization indexes are obviously dislocated and distorted. Simply, when the FCTP is clamped by enough tightening torque (which ensures it is under good constraint conditions), then the damage localization indexes become easy and clear to identify.

2. Impact assessment of the varied excitation levels.

Firstly, four different excitation levels are set up, namely 200 N, 490 N, 1100 N, and 1600 N, and each of them is used to excite the FCTP in the resonance state. Then, the same scanning parameters used in Section 5-list 1 are employed to complete modal shape scanning measurements. Table 4 lists the 4th curvature modal shapes and the 4th damage localization indexes of the FCTP measured under the varied excitation levels, which have some slight influences on natural frequency. For instance, with the increase in the excitation level, the 3rd natural frequency of the FCTP reduces slightly, which indicates a soft nonlinear stiffness characteristic. While the excitation level is small, i.e., it is equal to 200 N or 490 N, it will lead to unsatisfactory curvature modal shape results (e.g., fuzzy damage localization indexes). And with the increase in excitation level, the curvature modal shapes and the damage localization indexes become clearly identified. But when the excitation energy is increased to 1600 N, the curvature modal shapes and the damage localization indexes appears unchanged compared with the ones under the excitation level of 1100 N. So, in order to improve the test accuracy of modal shape results, we need use a sufficient excitation level to excite the FCTP in the resonance state but also should avoid too much excitation energy, because it may cause other negative effects, including geometric nonlinearity, too much local vibration, etc.

3. Impact assessment of the varied scanning rates.

In order to assess the impact of laser scanning rate on test accuracy of the curvature modal shapes results, we firstly choose three different scanning rate values, namely 3 mm/s, 10 mm/s, and 20 mm/s, and then set excitation levels as 1100 N and the adjacent row spacing as 10 mm to finish the modal shape scanning test. Table 5 gives the 4th curvature modal shapes of the FCTP measured under the above different scanning rates. Table 6 gives the 4th damage localization indexes of the FCTP measured under the varied laser scanning rates. It can be found out that, when the scanning rate is 3 mm/s, the curvature modal shapes and the damage localization indexes become clearly identified. But with the increase in scanning rate, it will lead to unsatisfactory shape results (e.g., fuzzy damage localization indexes).

This negative phenomenon can be explained by the selection criterion of the laser scanning rate proposed. Here we firstly assume the diameter of the laser point is about 3 mm and the length of the FCTP is 200 mm. According to the selection criterion of the laser scanning rate and extraction principle of modal shape data [30], we obtain the following selection criterion of η.

(16) $$\eta =\frac{l}{11\cdot v\cdot \Delta t}\geq 1$$

where Δt is the time width, we can set Δt as 1 s [30].

Then, substituting the used scanning rate values of 3 mm/s, 10 mm/s, and 20 mm/s into Equation (16), we can obtain the selection criterion η as 6.06, 1.82, and 0.91, correspondingly. According to the selection criterion of laser scanning rate, only when η>1 can the curvature modal shape results be correctly obtained by way of the laser linear scanning method. Therefore, the scanning rate of 20 mm/s has not met the selection criterion.

## 6. Conclusions

In the paper, the damage location detection of the FCTP is studied using a combination of theoretical and experimental approaches. Based on the CMSSM and the two-dimensional five-spot-tripling surface smoothing method, a quantitative index for damage location detection of the FCTP, i.e., the damage localization index, can be derived. Then, the principle of damage localization is clarified, and the specific damage location detection procedures of the FCTP based on the laser scanning method are also proposed. Finally, the influence of parameters such as boundary constraints, excitation level, and laser scanning rate on the damage detection results is also discussed, and the conclusions are as follows:

1. The TC500 fiber/epoxy composite plate is utilized as an experimental object, and the damage identification experiment is performed based on the established laser scanning vibration testing system. The experimental results shows that the ADLI is significantly higher near the fiber break points than in other parts, proving that this method can indeed accurately detect the fiber breakage position of this FCTP.
2. The boundary condition has a large influence on curvature modal shapes and damage localization indexes of the FCTP, so it must be clamped to ensure it is in a good constraint state by enough tightening torque. In this way, the damage localization indexes become clear and easy to identify through the CMSSM.
3. When the excitation level is low, it will lead to unsatisfactory curvature modal shape results (e.g., fuzzy damage localization indexes), and with the increase in excitation level, the curvature modal shapes and the damage localization indexes become clearly identified. So, to improve the accuracy of the test, we need use a sufficient excitation level to excite the tested FCTP in the resonance state but also need to avoid too much excitation energy.
4. The laser scanning rate has effects on curvature modal shapes and damage localization indexes. It can be found out that when the scanning rate is 3 mm/s, the curvature modal shapes and the damage localization indexes become clearly identified. But with the increase in scanning rate, it will lead to unsatisfactory curvature modal shapes and damage localization indexes results, and we can choose an appropriate laser scanning rate by referring to the proposed selection criterion.
5. The two-dimensional laser scanning vibration system is used to accurately detect damage locations of two-dimensional plates. The accuracy of the damage location of the shell structure needs to be further verified. In the future, the application of the three-dimensional testing system for shell structure damage positioning will continue to be studied. In addition, the experimental results show that this method can accurately locate symmetrically orthogonally laid composite materials. The accuracy of using this method to detect the fiber breakage location of woven composite materials will also be studied.

## Figures and Tables

**Table 1:** The first 5 natural frequencies, modal shapes, curvature modal shapes, and smooth curvature modal shapes of the FCTP obtained in the laser scanning test.

Modal Order	Natural Frequency	Modal Shape	Curvature Modal Shapes	Smooth Curvature Modal Shapes
1	31.0 Hz	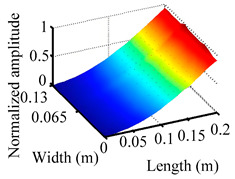 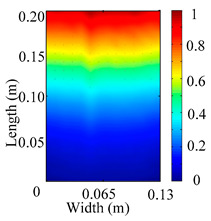	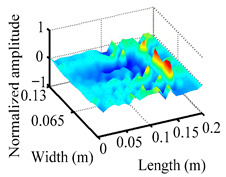 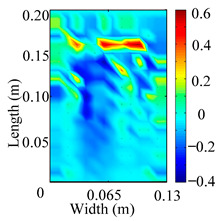	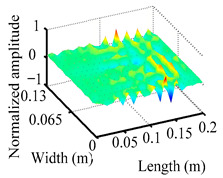 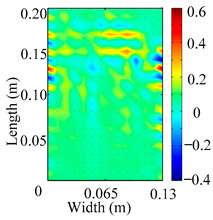
2	175.5 Hz	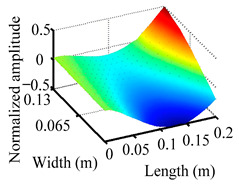 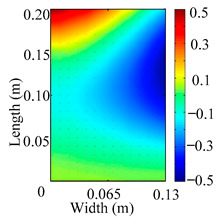	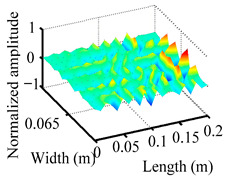 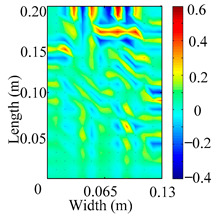	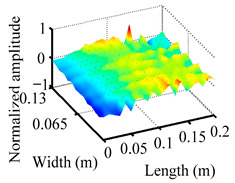 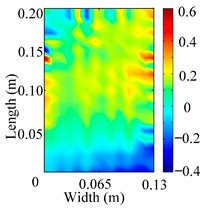
3	193.5 Hz	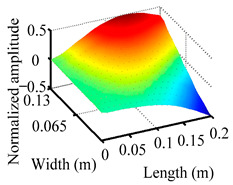 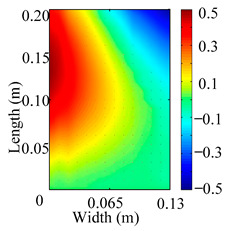	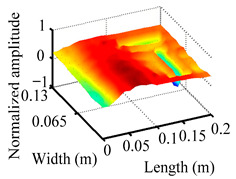 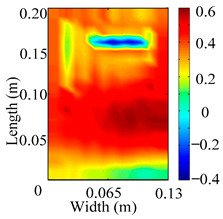	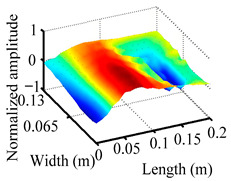 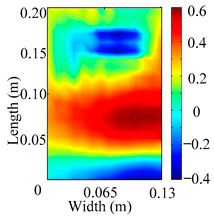
4	499.5 Hz	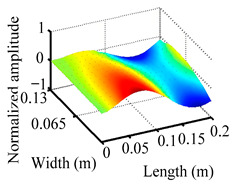 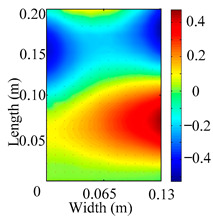	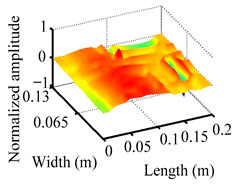 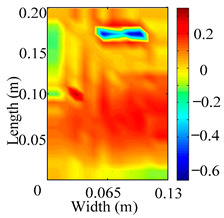	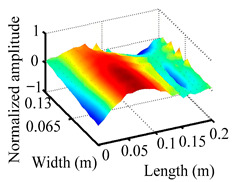 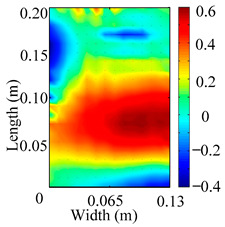
5	580.5 Hz	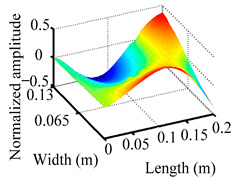 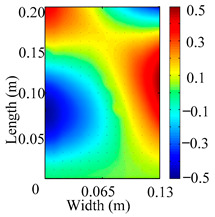	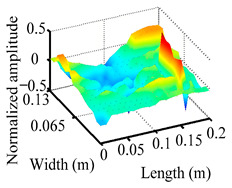 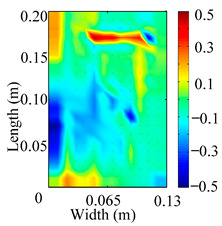	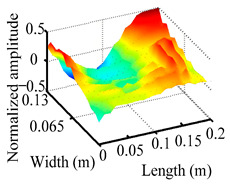 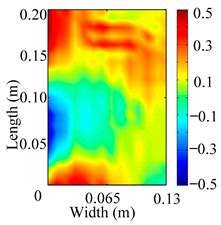

**Table 2:** The 3rd curvature modal shapes of the FCTP measured under the three different constraint boundaries.

Constraint Boundary I (5 Nm Tightening Torque)	Constraint Boundary II (30 Nm Tightening Torque)	Constraint Boundary III (50 Nm Tightening Torque)
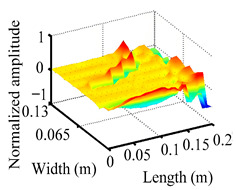 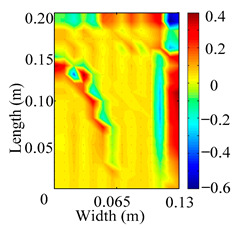	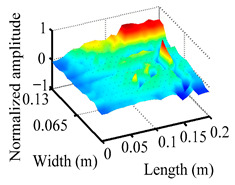 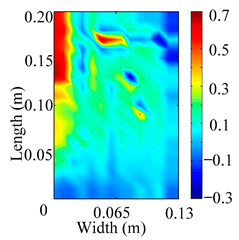	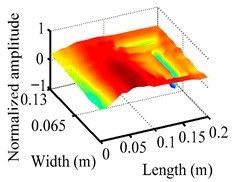 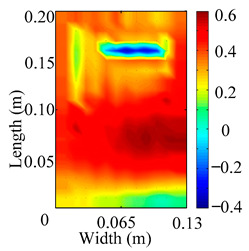

**Table 3:** The 3rd damage localization indexes of the FCTP measured under the three different constraint boundaries.

Constraint Boundary I (5 Nm Tightening Torque)	Constraint Boundary II (30 Nm Tightening Torque)	Constraint Boundary III (50 Nm Tightening Torque)
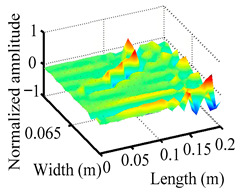 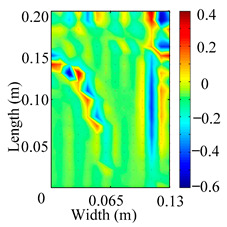	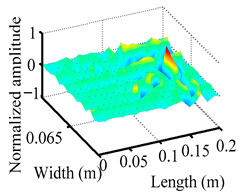 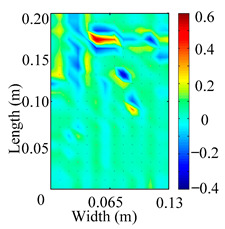	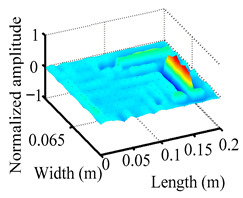 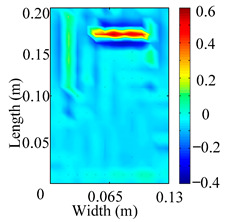

**Table 4:** The 4th curvature modal shapes and the 4th damage localization indexes of the FCTP measured under the different excitation levels.

Excitation Level	Natural Frequency (Hz)	Curvature Modal Shapes	Damage Localization Indexes
200 N	501.0	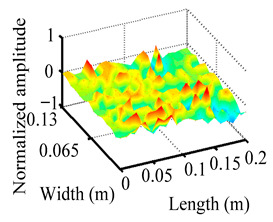 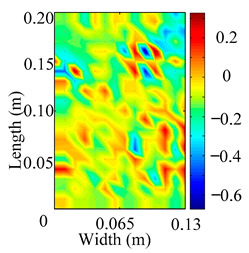	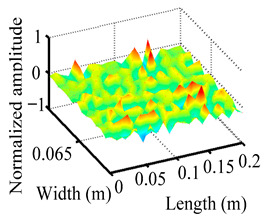 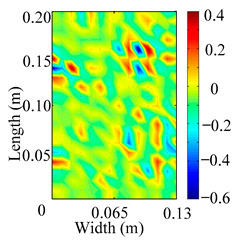
490 N	500.2	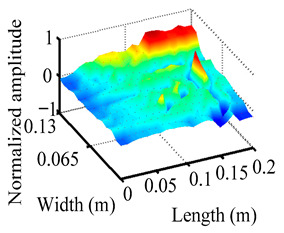 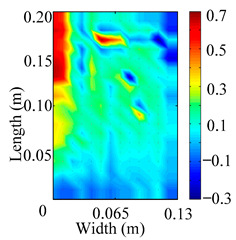	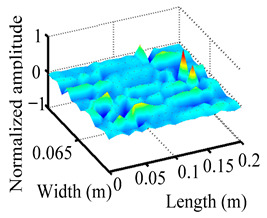 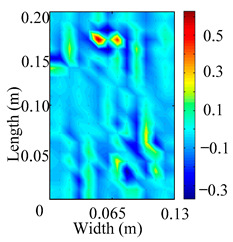
1100 N	499.5	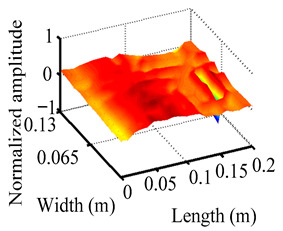 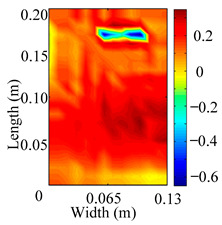	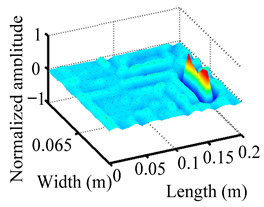 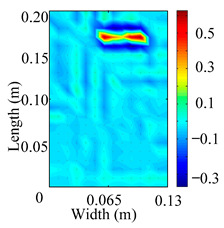
1600 N	498.3	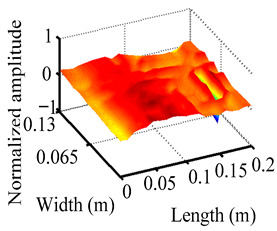 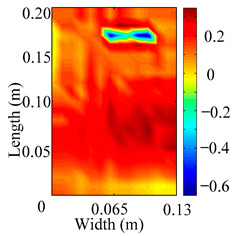	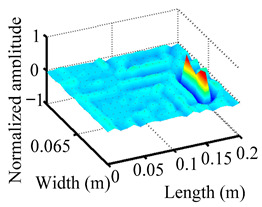 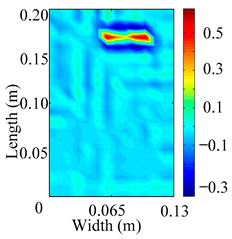

**Table 5:** The 4th curvature modal shapes of the FCTP measured under the varied laser scanning rates.

Laser Scanning Rate of 3 mm/s	Laser Scanning Rate of 10 mm/s	Laser Scanning Rate of 20 mm/s
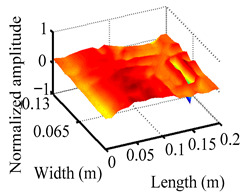 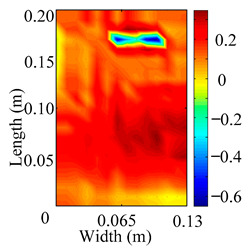	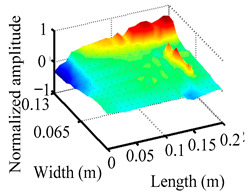 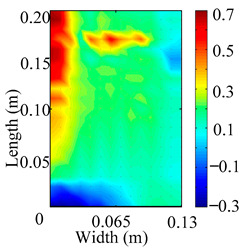	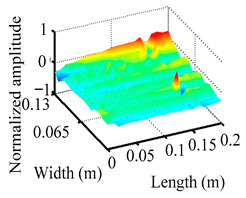 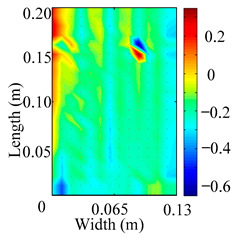

**Table 6:** The 4th damage localization indexes of the FCTP measured under the varied laser scanning rates.

Laser Scanning Rate of 3 mm/s	Laser Scanning Rate of 10 mm/s	Laser Scanning Rate of 20 mm/s
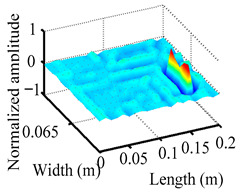 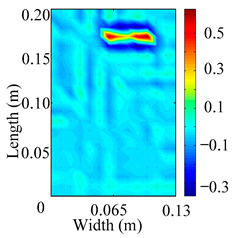	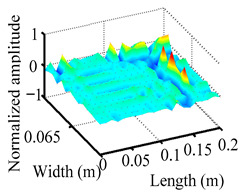 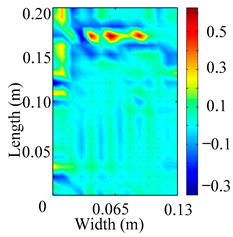	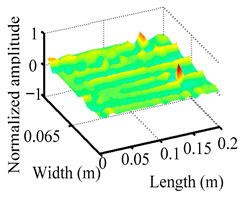 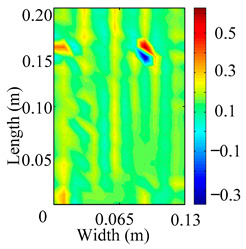

## Data Availability

The original contributions presented in this study are included in the article. Further inquiries can be directed to the corresponding author.

## Abbreviations

The following abbreviations are used in this manuscript:

CMSSM	Curvature Modal Shape Scanning Method
FCTP	Fiber-reinforced Composite Thin Plate
ADLI	Average Damage Localization Index
